# Quadruple Theories Based Determinants and their Causal Relationships Affecting the Adoption of Community Cloud in Saudi HEI

**DOI:** 10.1155/2022/2382535

**Published:** 2022-04-28

**Authors:** Nouf S. Aldahwan, Muhammed S. Ramzan

**Affiliations:** ^1^Department of Information System, King Abdulaziz University, Jeddah, Saudi Arabia; ^2^Department of Information System, King Khaled University, Abha, Saudi Arabia

## Abstract

The higher education institutions (HEIs) are adopting the new modern cloud computing technique rapidly due to its cost effectiveness, efficient and productive feature. Though cloud computing technology is beneficial to educational sector, it is important to assess their economic benefits, technical, organizational, environmental appropriateness and potential obstacles before adopting the new technology. There are four evaluating theory for adopting the cloud computing technology which are the Technology Organization Environment (TOE), the Technology Acceptance Model (TAM), the Diffusion of Innovation (DOI), and the Institutional (INT). This study has developed a new adoption framework for accepting cloud computing technology for HEIs of Saudi by integrating the above mentioned theories. This framework is unique from others because no research has been conducted yet on the adoption of community cloud at the organizational level considering the four theory simultaneously. This research has developed 25 hypotheses on the adoption of community cloud computing in HEIs and analyzed those hypotheses using SPSS statistical analysis software. The reliability of the data was tested by utilizing composite reliability and Cronbach's alpha method. This study have introduced an innovative approach and framework to understand the adoption of the community cloud which will help the decision-makers to build strategies in their organizations for effective adoption of community cloud services.

## 1. Introduction

Several enterprises are now adopting the new cloud computing technology for its cost effectiveness, efficient and productive feature. The cost savings are achieved as the facilities are also now part of a “rent model” rather than being “centrally hosted.” [1]. Cloud computing has multiple distinct features such as multi-tenancy, pooling of collective resources, greater diversity, competitive resource provision, and facility-based charging, which vary from conventional service computing. Everywhere, it has network connectivity and is seeded and self-organizing.

Saudi universities depend mainly on traditional information and communications technology (ICT) for supporting their educational services [2]. Each university has their own computing facilities that comprise of hardware, software, network, and storage to meet their needs and providing better services. Recently, they have begun adopting cloud computing technology which needs a huge investment. It is not a viable option for individual institutions to adopt cloud services by employing many service providers, deploying various cloud models and manage own computing facilities comprising of hardware and software. This will cause wasting time and more expenses for the organizations. In addition, HEIs are facing many challenges in adopting cloud computing such as data security and privacy [3, 4], resource scheduling [4]. Instead of individual cloud computing, the Community cloud computing will be the best possible solution of the above-mentioned adopting problem. In Community cloud computing, organizations can share the benefits as well as the expenses for it. Concerning the community model to gain an advantage through risk perception, shared resources [5], and cost savings [6].

All government universities in Saudi follow to the same regulations and are funded by the government. They depend mainly on the Saudi ministry of education for providing the requested budget for its ICT operations. So, Saudi government can impose all Saudi universities to adopt community cloud computing technology which will be controlled by Ministry of Saudi Higher Education. It can be used by the HEIs higher management and the end-user (teachers and students). As a result, it is important to understand about the factors that influence Saudi HEIs' adoption of the community cloud.

A community cloud is developed when multiple organizations require similar infrastructure and wish to exchange the same. By sharing the infrastructure, organizations thus realize the benefits of cloud computing. The costs of the community cloud users will be less than an individual or one tenant because it spreads over the multiple users in community cloud. The community cloud is, therefore, cost effective although it provides a high level of privacy, policy compliance and security [7].

This study intends to contribute to the body of information surrounding technology adoption. It will provide empirical evidence in the field of community cloud and provide a comprehensive model that integrates four major adoption theories (quadruple theories): the technology-organizational-environmental (TOE) framework, the technology acceptance model (TAM), the diffusion of innovations (DOI), and the institutional (INT) theory to fill the gap in previous literature. By following the theories of acceptance at the organizational level, this research model is based on the TOE, TAM, DOI and INT theories that are in line with the objectives of this report. The goals of this study are to assess major factors contributing to adoption of community cloud in Saudi HEIs and support the decision-makers (management and technical managers) to leverage community cloud in HEIs.

The purpose of this research will be achieved by analyzing the viewpoints of IT and telecommunications experts, clients of cloud computing-supporting devices, the scope of community cloud adoption in Saudi Arabia, the driving factors, and current obstacles to community cloud adoption. The first section of this paper provides an introduction, while the second section contains theoretical background. The research framework and hypothesis will be present in section 3. Result will be explained in Section 4 to meet the research's goals. The fifth section is Discussion. Finally, the conclusion.

## 2. Theoretical Background

### 2.1. Cloud Computing in Saudi Higher Education

With the diffusion of information technology and telecommunication, higher education requires to keep up with development and evolution. The Ministry of Education coordinates Saudi Arabia's education system, which now includes 25 public and 8 private universities [8]. Since many institutions throughout the world are moving toward gaining e-learning services in their colleges, cloud computing has been implemented in Saudi Arabia for various universities.

Al-Ruithe [9] conducted an empirical study and a survey to examine the present situation of cloud computing adoption in the Kingdom of Saudi Arabia. It has demonstrated that Saudi Arabia is still evaluating technology, with few adoptions, and certainly not at the government level. Future study will focus on analyzing the remaining data from the questionnaire, with a special focus on the adoption obstacles.

Alamri and Qureshi [10] have addressed the need to use cloud computing in higher education in Saudi Arabia to improve the level and find solution for the obstacles facing the learning process. It has also addressed the problems, which they face during the education process. It has provided a roadmap to teachers and students for applying cloud computing in higher education. Firstly, the study introduces the cloud computing courses to prepare students to adopt in cloud computing. Secondly, supports the use of cloud infrastructure for sharing virtual classes, online lectures. Finally, it is measuring cloud computing technology efficiency for all disciplines.

Noor [11] proposed a two-dimensional research framework for motivators and inhibitors to explore the use and approval of cloud computing technology in Saudi Arabia which relied on empirical analysis of numerous data collected from five universities in Saudi Arabia: King Abdul-Aziz University, King Saud University, Imam Muhammad ibn Saud Islamic University, Taibah University, and Umm al-Qura University.

According to his findings, network access and self-service are the two most important factors for Saudi cloud users to use cloud computing, with 51 percent and 23 percent, respectively. Availability, dependability, security, compliance, and privacy are the top five obstacles. This can assist decision-makers when it comes to cloud computing adoption.

In addition, Smart suitcase is a cloud service provided by Princess Noura Bint Abdul Rahman University in collaboration with Microsoft and Saudi Telecommunication Company (STC). Smart suitcases enable effective and efficient communication, information, and data exchange between university faculties and college students [12].

Microsoft has agreed to give a cloud storage service to King Saud University staff. Many capabilities are included in the cloud storage service, including secure and easy file sharing, utilizing a web browser to edit files in Microsoft Office, synchronizing data on your PC's hard disk, and easily accessing online files stored on cellphones or PCs [13].

### 2.2. Community Cloud Computing

The community cloud developed from mixing its use scenarios with network computing models, digital ecosystem principles, autonomous management from autonomous computing, and green computing sustainability. It fast emerging as a shifting model in many fields, such as analysis and finance. Many virtual communities developed with community cloud computing technology exchange their resources such as scientific knowledge and software applications for many fields [14].

Many factors influence community cloud applications, including ease of use, quality of service, security, cost, and adequate resource [15].

Nonetheless, as compared to other sectors, such as government and business, the use of community cloud in higher education institutions is still in its early phases.

The article by Aldahwan and Ramzan [16], studiedcontributes to the field of community cloud computing research by offering a comprehensive review of related trends in concepts, methodology, research framework, architecture, model, and future research directions.

Based on earlier studies, there is a problem to determine the factors impacting the adoption of community clouds in higher education institutions from a decision-makers' perspective. As a result, more research is needed to determine how important aspects impact the adoption of community cloud in HEIs at the organizational level.

### 2.3. Technology Adoption Theories

Adoption processes are crucial to the decision-maker (adopter) or the undertaking unit for implementing a new service or concept [17]. According to [18], the acceptance decision of a specific object, in a particular context, by an individual is affected by multiple variables. The HEI is the individual in the present analysis, while cloud computing adoption is the object. After reviewing related studies, it was discovered that several studies have focused on the impact of cloud computing adoption at an individual level ([19–23]); however, To the authors' best knowledge, there was a lack of sufficient research on community cloud computing at the organizational level [24–26]. The proposed framework combines the four theories used for as discussed by [22–24, 27–33].  Table 1 shows the cloud computing adoption in HEIs.

#### 2.3.1. Technology–Organization–Environment (TOE) Theory

It provides a thorough explanation of the likelihood of a certain company adopting new technology by including technology-organization, environment, and socio-cultural variables. According to the TOE paradigm, both the limitations that function as barriers and the opportunities that work as accelerators for technological advancement have these three characteristics [34].

#### 2.3.2. Diffusion of Innovation (DOI) Theory

The theory of DOI, was invented by Everett Roger [35]. Much research on how individuals accept new ideas have made extensive use of this technique. The primary goal of the DOI theory is to aid all organizations and individuals, regardless of whether they embrace or reject the advances [36].

#### 2.3.3. Technology Acceptance Model (TAM)

It is the information system theory that is frequently utilized when attempting to explain why a person accepts a particular type of information system. It is a model that is moving in the correct way when one considers its efforts to handle the problem of underutilized structures. According to the original TAM, there were five factors to consider: perceived ease of use; perceived advantage; attitude toward using the system; intent to do activities; and actual usage [37].

#### 2.3.4. Institutional Theory (INT)

It discusses how corporations act as institutions in influencing the behaviors and perspectives of the people who work for them [38]. The theory provides insights on the significance of organizational structure and work. It is observed that company decisions are influenced not just by rational production goals, but also by social and cultural considerations [39].

Several current studies have ignored empirical results on the use of cloud computing in HEIs [24, 40]. More research on community cloud adoption at HEIs, as well as a complete description of the factors that influence this process, is needed due to a lack of evidence at the organizational level.  Table 1 summarizes the previous cloud adoption literature in higher education institutions, which includes the TOE, DOI, TAM, and INT models. This study is founded on organizational level theories (i.e., TOE, INT, and DOI), which are in line with the aim of this study.

**Figure 1:**
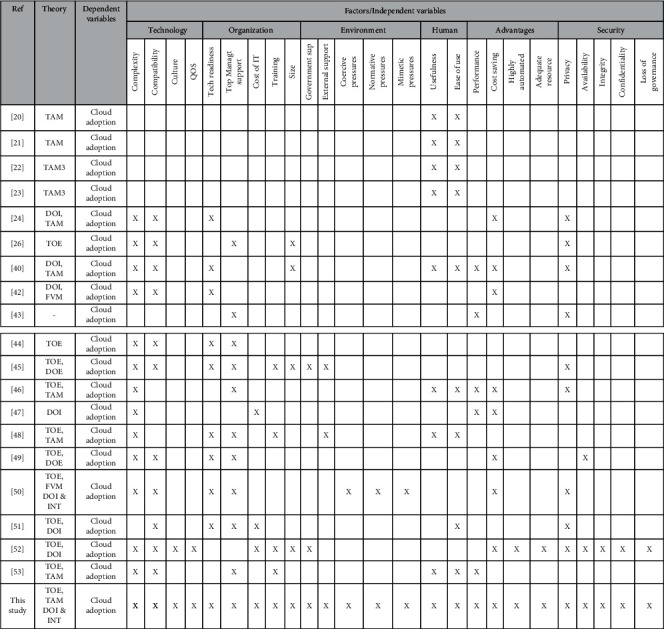
Mapping matrices build from quadruple theories (TOE, TAM, DOI and INT) theories.

Mapping matrix of past adoption theories and literature-relevant factors, as shown in Figure 1.

## 3. Research Framework & Hypotheses

**Figure 2:**
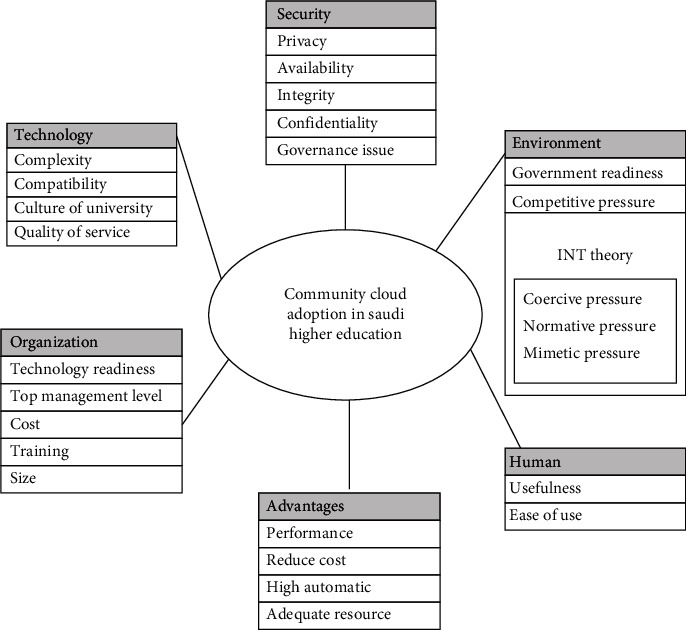
Research Framework.

The TOE framework is a realistic model that can be used as a foundation for the proposed framework with additional contexts to this study. The TOE framework aims to clarify the innovation process at the organization level, considering the three contexts affecting the adoption of innovation in a company - technology, organization, and environment. Moreover, DOI (innovation) theory also taught in mind to understand the diffusion of innovation. The INT theory describes insight into allowing institutional constraints that may influence technology adoption and innovation, and it implies that organizations are subject to three types of pressures: pressurized/coercive, normative, and mimetic. One of the most popular models for technology adoption is TAM, with two essential factors impacting an individual's intent to use the new technology. The research framework contains the following context, as shown in Figure 2.

Our research will be guided by the hypotheses listed below, which are based on our suggested conceptual framework model.

H1: Competitive pressure positively influences community cloud adoption in HEIs.

H2: Compatibility positively influences community cloud adoption in HEIs.

H3: University Culture positively influences in the community cloud adoption in HEIs.

H4: Perceived benefits of Quality of Service positively influences in the community cloud adoption in HEIs.

H5: Technology readiness positively affects community cloud adoption in HEIs.

H6: Top management support positively affects community cloud adoption in HEIs.

H7: Cost of IT operations negatively influences community cloud adoption in HEIs.

H8: Training for staff positively influences community cloud adoption in HEIs.

H9: University size positively influences community cloud adoption in HEIs.

H10: Government support positively influences community cloud adoption in HEIs.

H11: External support positively influences community cloud adoption in HEIs.

H12: Coercive pressures positively influence community cloud adoption in HEIs.

H13: Normative pressures positively influence community cloud adoption in HEIs.

H14: Mimetic pressures positively influence community cloud adoption in HEIs.

H15: Perceived usefulness positively influences people's intention to use a community cloud.

H16: Perceived ease of use positively influences people's intention to use a community cloud.

H17: Perceived benefits of performance positively influence community cloud adoption in HEIs.

H18: Cost saving positively influences community cloud adoption in HEIs.

H19: Highly automated positively influences community cloud adoption in HEIs.

H20: Adequate resource positively influences community cloud adoption in HEIs.

H21: Privacy risks negatively influences community cloud adoption in HEIs.

H22: Availability positively influences in community cloud adoption in HEIs.

H23: Lower degree of integrity negatively influences community cloud adoption in HEIs.

H24: Lower degree of confidentiality negatively influences community cloud adoption in HEIs.

H25: Loss of governance negatively influences community cloud adoption in HEIs.

## 4. Result

### 4.1. Reliability and Validity Test

**Figure 3:**
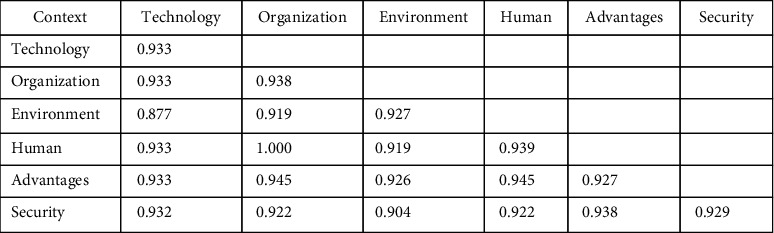
Fornell–Larckers criterion analysis construct.

The composite reliability and Cronbach's Alpha were utilized for assessing the internal consistency of the model by following the work of Hair Jr, Hult [54]. “ The constructs' validity was evaluated using “average variance extracted (AVE)“ and “cross-factor loadings.” The findings of the stated constructs' reliability and validity were represented in Figure 3.

When the value of Cronbach's Alpha is greater than 0.7, it indicates that the survey data and the constructs' reliability ensure satisfactory internal consistency [54]. The data revealed that composite reliability and Cronbach's alpha are both greater than the suggested value of 0.7 for all identified components, indicating that they are more reliable than the recommended level and all items are statistically significant at the 0.01 level. In addition, an acceptable AVE value for each construct is 0.50 [47]. As shown in Table 2, the scale item validity was greater than 0.5 which is above the threshold value. Following convergent validity validation, discriminant validity is the next step. The constructs' “discriminant validity” [55] was determined by examining their relationships. Figure 3 shows that the square root of AVE for specified constructs was higher than the correlation co-efficient for other latent constructs. As a result, in the evaluation of the measurement model [55], “convergent and discriminant validity” was approved. As a result of the foregoing evaluations, the validity and reliability of the measurement model's constructs have been acknowledged, and they satisfy the recommended values as given in Table 2.

### 4.2. Hypothesis Analysis

The mentioned hypotheses have been investigated to see whether they may be linked to one another. The null hypothesis states that there is no statistically significant relationship between two research variables. The alternate hypothesis is that there is a statistically significant relationship between two of the research variables. Hypotheses describing the link between two study variables has been tested. The test of the hypotheses is shown in Table 3.

- The null hypothesis states that there is no statistically significant relationship between two research variables
- The alternative hypothesis states that two of the research variables have a statistically significant relationship

According to SPSS program results, the null hypothesis will be accepted if the P-value is greater than 0.05. In Table 3, the P-value is less than 0.05 which indicates that the null hypothesis does not satisfy the acceptance criteria and it is rejected. The alternate hypothesis will be rejected if the P-value is greater than 0.05. In Table 3, the P-value is less than 0.05 which indicates that the alternate hypothesis is accepted. After analyzing the data, it is evident that there is a significant statistical relationship between two research variables.

The correlation coefficient between dependent variable and all independent variables shows that the correlation coefficient is statistically significant because the *p*-value (Sig.) is less than 0.05 as shown in Table 3. There prevails a substantial relationship between dependent variable and independent variables.

## 5. Discussion

### 5.1. Managerial and Theoretical Implications

This research provides a significant contribution to the body of knowledge of community cloud research and the adoption criterion of it in the higher educational institutions.

- To the authors' best knowledge, the existing literature lacks in examining the influencing factors of the adoption of community cloud at the organizational level in HEIs by incorporating four dominant theories. This study has integrated the TOE, TAM, DOI, and INT theory which is very important for the implementation of community cloud
- This significant study is intended to serve as a starting point for subsequent research on the impact of community cloud adoption in higher education institutions, which will be carried out in the future
- The study can be used by HEI decision-makers to determine whether to embrace community cloud services in their institutions, by tying their knowledge to the study's goals and deciding before implementing educational community cloud-based projects
- The product model can be applied to comparable technology adoption scenarios as well as other industries
- The proposed study's findings may provide significant information to industry practitioners and community cloud service providers that can be used to manage and sell community cloud initiatives

### 5.2. Validation and Methodological Implications Approaches

We recommend that the proposed framework should be tested by a pilot study at multiple research universities (RUs). The theoretical approach obtained from this critical study should be implemented and tested at a few higher education institutions to test the suggested framework. As a result, higher education institutions can adopt the created findings or outcomes of this research. Furthermore, empirical experiments on the model are required to validate the connection between adoption theories.

### 5.3. Future Research and Limitations

The future research can be conducted to evaluate the developed framework and assess its descriptive and analytical capabilities by validating and adopting it to several HEIs. To enhance the performance and provide a good explanation for the independent variables, future studies should critically examine other relations which are not covered by this model. Future research can investigate the impact of community cloud and how it affects HEIs.

The research can be extended in the future to see how the target population's adoption changes over time and how the determinant factors change. This research might be carried out in various countries to compare the important factors among them.

## 6. Conclusion

The pre-adoption stage of a conceptual framework based on TOE, TAM, DOI, and INT theories by Saudi HEIs is discussed in this research. This framework aids in finding the characteristics that drive community cloud adoption in HEIs that have not yet adopted community cloud services. It is widely believed that HEIs must adopt community cloud services not just to save money, but also to increase efficiency and effectiveness. This research provides a significant knowledge about the influence of community cloud adoption in higher educational institutions. It will also deliver useful information to institutions and academicians who are interested to utilize the findings of this research in real-life practice. This comprehensive study will also serve as a hypothetical framework for future research on the impact of community cloud adoption in the educational sector at HEIs.

## Figures and Tables

**Table 1:** Cloud computing adoption in HEIs.

Theory	Methodology		
—	Survey	USA	[19]
TAM	Survey	Romania	[20]
	Quantitative method, survey	Thailand	[21]
TAM3	An empirical study, quantitative method, survey	Saudi Arabia	[22]
	Virtual computing lab and focus groups	USA	[23]
DOI, TAM	Quantitative research, survey	Quantitative research, survey	[24]
	Conceptual model, pilot study	Africa	[40]
TOE	An empirical study, quantitative method, survey	Saudi Arabia	[26]
	Conceptual model, survey	India	[41]
—	Interviews.	Oman.	[42]
DOI. FVM	Conceptual model	Oman	[43]
—	Questionnaire	Saudi Arabia	[44]
TOE, TAM	Conceptual model	Bangladesh.	[45]
	Quantitative methods, survey	Saudi Arabia	[46]
	Conceptual model, survey	India	[47]
TOE, DOI	Survey	Saudi Arabia	[48]
	Conceptual model	Malaysia.	[49]
	Conceptual model, survey	Ethiopia,	[50]
	Conceptual model, survey	Pakistan	[51]
DOI	Empirical study, focus group	Taiwan	[52]
TOE, FVM, DOI, INT	Conceptual model	Malaysia	[53]
This paper	Quantitative method, survey	Saudi Arabia	

**Table 2:** Constructs' reliability and validity.

Factors	Cronbach's alpha (CA>0.6)	Composite reliability (CR>0.7)	AVE (CR>0.5)
Complexity	.953	.964	.871
Compatibility	.927		
Culture of university	.927		
Quality of service	.927		
Technology readiness	.928	.973	.880
Top management level	.943		
Cost	.944		
Training	.944		
Size	.932		
Government readiness	.928	.968	.860
Competitive pressure	.928		
Coercive pressure	.927		
Normative pressure	.928		
Mimetic pressure	.928		
Usefulness	.927	.937	.881
Ease of use	.951		
Performance	.927	.960	.860
Reduce cost	.927		
High automatic	.928		
Adequate resource	.928		
Privacy	.930	.969	.863
Availability	.929		
Integrity	.928		
Confidentiality	.928		
Governance issue	.931		

**Table 3:** Correlation coefficient between (dependent variables) and (independent variables).

Hypothesis	*p*-value	Standard error	Pearson correlation	Decision
H1: Complexity negatively influences community cloud adoption in HEIs.	*p ≤0.0*	0.47	0.949	Support
H2: Compatibility positively impacts community cloud adoption in HEIs.	*p ≤0.0*	0.49	0.946	Support
H:3 university culture positively impact in the community cloud adoption in HEIs.	*p ≤0.0*	0.41	0.962	Support
H4: Perceived benefits of quality of service positively impacts in the community cloud adoption in HEIs.	*p ≤0.0*	0.35	0.971	Support
H5: Technology readiness positively impact community cloud adoption in HEIs.	*p ≤0.0*	0.35	0.972	Support
H6: Top management support positively impacts community cloud adoption in HEIs.	*p ≤0.0*	0.38	0.942	Support
H7: Cost of IT operations negatively influences community cloud adoption in HEIs.	*p ≤0.0*	0.38	0.942	Support
H8: Training positively impacts community cloud adoption in HEIs.	*p ≤0.0*	0.38	0.963	Support
H9: University size positively influences community cloud adoption in HEIs.	*p ≤0.0*	0.46	0.944	Support
H10: Government support positively influences community cloud adoption in HEIs.	*p ≤0.0*	0.28	0.857	Support
H11: External support positively influences community cloud adoption in HEIs.	*p ≤0.0*	0.82	0.841	Support
H12: Coercive pressures positively influence community cloud adoption in HEIs.	*p ≤0.0*	0.28	0.839	Support
H13: Normative pressures positively influence community cloud adoption in HEIs.	*p ≤0.0*	0.30	0.810	Support
H14: Mimetic pressures positively influence community cloud adoption in HEIs.	*p ≤0.0*	0.69	0.891	Support
H15: Perceived usefulness positively influences people's intention to use a community cloud.	*p ≤0.0*	0.30	0.927	Support
H16: Perceived ease of use positively influences people's intention to use a community cloud.	*p ≤0.0*	0.46	0.951	Support
H17: Perceived benefits of performance positively influence community cloud adoption in HEIs.	*p ≤0.0*	0.49	0.946	Support
H18: Cost saving positively influences community cloud adoption in HEIs.	*p ≤0.0*	0.75	0.868	Support
H19: Highly automated positively influences community cloud adoption in HEIs.	*p ≤0.0*	0.81	0.846	Support
H20: Adequate resource positively influences community cloud adoption in HEIs.	*p ≤0.0*	0.79	0.853	Support
H21: Privacy risks negatively influences community cloud adoption in HEIs.	*p ≤0.0*	0.28	0.890	Support
H22: Integrity positively influences in community cloud adoption in HEIs.	*p ≤0.0*	0.49	0.945	Support
H23: Availability positively influences in community cloud adoption in HEIs.	*p ≤0.0*	0.42	0.960	Support
H24: Lower degree of confidentiality negatively influences community cloud adoption in HEIs.	*p ≤0.0*	0.48	0.948	Support
H25: Loss of governance negatively influences community cloud adoption in HEIs.	*p ≤0.0*	0.48	0.948	Support

## Data Availability

The primary and secondary data used to support the findings of this study are available from the corresponding author upon request.

## References

[1] Koch F., Assunçao M. D., Netto M. A. A Cost Analysis of Cloud Computing for Education.

[2] Alharthi A., Wills G., Alassafi M., Walters R., Alzahrani A. (2017). Critical success factors for cloud migration in higher education institutions: a conceptual framework. *International Journal of Intelligent Computing Research*.

[3] Kong W., Lei Y., Ma J. (2018). Data security and privacy information challenges in cloud computing. *International Journal of Computational Science and Engineering*.

[4] Birje M. N., Challagidad P. S., Goudar R. H., Tapale M. T. (2017). Cloud computing review: concepts, technology, challenges and security. *International Journal of Cloud Computing*.

[5] Qasim A., Sadiq A., Kamaludin A., Al-Sharafi M. E-learning models: The e_ectiveness of the cloud-based E-learning model over the traditional E-learning model.

[6] Aldahwan N. S., Saleh M. S. Developing a framework for cost-benefit analysis of cloud computing adoption by higher education institutions in Saudi Arabia.

[7] Rao C. C., Leelarani M., Kumar Y. R. (2013). Cloud: computing services and deployment models. *International Journal of Engineering and Computer Science*.

[8] Abubakar I. R., Al-Shihri F. S., Ahmed S. M. (2016). Students’ assessment of campus sustainability at the University of Dammam Saudi Arabia. *Sustainability*.

[9] Al-Ruithe M., Benkhelifa E., Hameed K. (2017). Current State of Cloud Computing Adoption - An Empirical Study in Major Public Sector Organizations of Saudi Arabia (KSA). *Procedia Computer Science*.

[10] Alamri B., Qureshi M. (2015). Usability of cloud computing to improve higher education. *International Journal of Information Technology and Computer Science*.

[11] Noor T. H. (2016). Usage and technology acceptance of cloud computing in Saudi Arabian universities. *International Journal of Software Engineering and Its Applications*.

[12] E-services of Princess Nourah bint Abdulrahman University *Smart Suitcase*.

[13] E-transaction and Communication of King Saud University *Cloud Storage*.

[14] Marinos A., Briscoe G. Community cloud computing.

[15] Aldahwan N. S., Ramzan M. S. (2021). Factors Affecting the Organizational Adoption of Secure Community Cloud in KSA. *Networks*.

[16] Aldahwan N. S., Ramzan M. S. (2022). A Descriptive Literature Review and Classification of Community Cloud Computing Research. *Scientific Programming*.

[17] Rogers Everett M. (1995). *Diffusion of innovations*.

[18] Li Y.-H. An empirical investigation on the determinants of e-procurement adoption in Chinese manufacturing enterprises.

[19] Ashtari S., Eydgahi A. (2017). Student perceptions of cloud applications effectiveness in higher education. *Journal of Computational Science*.

[20] Militaru G., Purcarea A. A., Negoiţă O. D., Niculescu A. Examining Cloud Computing Adoption Intention in Higher Education: Exploratory Study.

[21] Bhatiasevi V., Naglis M. (2016). Investigating the structural relationship for the determinants of cloud computing adoption in education. *Education and Information Technologies*.

[22] Almazroi A. A., Shen H., Teoh K. Cloud for e-Learning: Determinants of Its Adoption by University Students in a Developing Country.

[23] Behrend T. S., Wiebe E. N., London J. E., Johnson E. C. (2011). Cloud computing adoption and usage in community colleges. *Behaviour & Information Technology*.

[24] Sabi H. M., Uzoka F. E., Langmia K., Njeh F. N., Tsuma C. K. (2018). A Cross-Country Model of Contextual Factors Impacting Cloud Computing Adoption at Universities in Sub-Saharan Africa. *Information Systems Frontiers*.

[25] Dahiru A. A., Bass J. M., Allison I. K. Cloud Computing Adoption in Sub-Saharan Africa: An Analysis Using Institutions and Capabilities.

[26] Tashkandi A. N., Al-Jabri I. M. (2015). Cloud computing adoption by higher education institutions in Saudi Arabia: an exploratory study. *Cluster Computing*.

[27] Oliveira T., Martins M. F. (2011). Literature review of information technology adoption models at firm level. *Electronic Journal of Information Systems Evaluation*.

[28] Kraemer K. L., Dedrick J., Melville N. P., Zhu K. (2006). *Global e-Commerce: Impacts of National Environment and Policy*.

[29] Lin H. F., Lin S. M. (2008). Determinants of e-business diffusion: A test of the technology diffusion perspective. *Technovation*.

[30] Dwivedi Y. K., Papazafeiropoulo A., Ramdani B., Kawalek P., Lorenzo O. (2009). Predicting SMEs’ adoption of enterprise systems. *Journal of Enterprise Information Management*.

[31] Mohammed F., Ibrahim O., Nilashi M., Alzurqa E. (2017). Cloud computing adoption model for e-government implementation. *Information Development*.

[32] Martins R., Oliveira T., Thomas M. A. (2016). An empirical analysis to assess the determinants of SaaS diffusion in firms. *Computers in Human Behaviour*.

[33] Oliveira T., Thomas M., Espadanal M. (2014). Assessing the determinants of cloud computing adoption: An analysis of the manufacturing and services sectors. *Information & Management*.

[34] Baker J. (2012). The Technology–Organization–Environment Framework. *Information System Theory*.

[35] Rogers E. M. (2010). *Diffusion of innovations*.

[36] Fagan M. (2001). Global information technology transfer: a framework for analysis. *Journal of Global Information Technology Management*.

[37] Lu J., Yu C. S., Liu C., Yao J. E. (2003). Technology acceptance model for wireless internet. *Internet Research: Electronic Networking Applications and Policy*.

[38] Chatterjee D., Grewal R., Sambamurthy V. (2002). Shaping up for e-commerce: institutional enablers of the organizational assimilation of web technologies. *MIS Quarterly*.

[39] Teo H. H., Wei K. K., Benbasat I. (2003). Predicting intention to adopt interorganizational linkages: an institutional perspective. *MIS Quarterly*.

[40] Sabi H. M., Uzoka F., Langmia K., Njeh K. (2016). Conceptualizing a model for adoption of cloud computing in education. *International Journal of Information Management*.

[41] Singh J., Mansotra V. (2019). Factors affecting cloud computing adoption in the Indian school education system. *Education and Information Technologies*.

[42] Alajmi Q. A., Kamaludin A., Arshah R. A., Al-Sharafi M. A. (2018). The Effectiveness of Cloud-Based E-Learning towards Quality of Academic Services: An Omanis’ Expert View. *E-Learning*.

[43] Alajmi Q., Arshah R. A., Kamaludin A., Sadiq A. S., Al-Sharafi M. A. A conceptual model of E-learning based on cloud computing adoption in higher education institutions.

[44] Alharthi A., Alassafi M. O., Walters R. J., Wills G. B. (2017). An exploratory study for investigating the critical success factors for cloud migration in the Saudi Arabian higher education context. *Telematics and Informatics*.

[45] Rahman M. M. (2020). Cloud computing in Bangladeshi higher educational institutions: influential factors and adoption model. *AIUB Journal of Business and Economics*.

[46] Tashkandi A., Al-jabri I. Cloud computing adoption by higher education institutions in Saudi Arabia analysis based on TOE.

[47] Gangwar H., Date H., Ramaswamy R. (2015). Understanding determinants of cloud computing adoption using an integrated TAM-TOE model. *Journal of Enterprise Information Management*.

[48] Alhammadi A., Stanier C., Eardley A. The Determinants of Cloud Computing Adoption in Saudi Arabia.

[49] Qasem Y. A. M., Asadi S., Abdullah R., Yah Y., Aan R. (2020). Applied sciences A Multi-Analytical Approach to Predict the Determinants of Cloud Computing Adoption in Higher Education Institutions. *Applied Sciences*.

[50] Hiran K. K., Henten A. (2020). An integrated TOE – DoI framework for cloud computing adoption in the higher education sector: case study of Sub-Saharan Africa. *International Journal of Systems Assurance Engineering and Management*.

[51] Tariq M. I., Tayyaba S., Rasheed H., Ashraf M. W. Factors Influencing the Cloud Computing Adoption in Higher Education Institutions of Punjab, Pakistan.

[52] Hwang B., Huang C., Yang C. L. (2016). Determinants and their causal relationships affecting the adoption of cloud computing in science and technology institutions. *Innovations*.

[53] Qasem Y. A. M. Mapping and Analyzing Process of Cloud-based Education as a Service (CEaaS) Model for Cloud Computing Adoption in Higher Education Institutions.

[54] Hair J. F., Hult G. T. M., Ringle C. M., Sarstedt M. (2021). *A Primer on Partial Least Squares Structural Equation Modeling (PLS-SEM)*.

[55] Fornell C., Larcker D. F. (1981). Structural Equation Models with Unobservable Variables and Measurement Error: Algebra and Statistics. *Journal of Marketing Research*.

